# Multidisciplinary Perspectives on Medical Decision-Making for Ostomy Surgery in Pediatric IBD: Themes from Focus Groups

**DOI:** 10.1007/s10880-024-10036-2

**Published:** 2024-08-14

**Authors:** Jennie G. David, Jennifer L. Dotson, Laura Mackner

**Affiliations:** 1 Nationwide Children’s Hospital, 700 Children’s Drive, Columbus, OH 43205 USA; 2 Department of Pediatrics, The Ohio State Wexner Medical Center, Columbus, OH USA; 3 Division of Pediatric Gastroenterology, Hepatology and Nutrition, Nationwide Children’s Hospital, Columbus, OH USA; 4 Center for Child Health Equity and Outcomes Research, Abigail Wexner Research Institute at Nationwide Children’s Hospital, Columbus, OH USA; 5 Center for Biobehavioral Health, Nationwide Children’s Hospital, Columbus, OH USA

**Keywords:** IBD, Multidisciplinary care, Ostomy, Surgery

## Abstract

Pediatric Inflammatory Bowel Disease (IBD) is a chronic illness where patients may undergo ostomy surgery. Medical decision-making (MDM) for ostomy surgery is complex for patients/families and multidisciplinary healthcare professionals (HCPs) alike, with current uncertainty about how multidisciplinary HCPs think about ostomy care to inform future interventions to facilitate equitable multidisciplinary care for patients. This study sought to understand pediatric IBD multidisciplinary HCPs’ perceptions regarding ostomy-related MDM and education. Multidisciplinary HCPs (e.g., gastroenterology medical providers, social workers, surgeons, and ostomy nurses) participated in semi-structured focus groups. Focus group data underwent qualitative analysis to identify themes. Three multidisciplinary focus groups were conducted, with *n* = 12 participants across all groups. Qualitative analysis identified three main themes, including (1) HCP perceptions of ostomies, (2) Patient/family-related factors, and (3) Professional roles and collaboration challenges. Ostomy surgery in pediatric IBD requires complex multidisciplinary MDM and education. Perspectives of multidisciplinary HCPs identified patient, HCP, and systems factors that may impact MDM for ostomy surgery. This work highlights nuances in MDM and education in IBD, and the critical role of ongoing research and improved standardized processes to coordinate multidisciplinary ostomy-related MDM and education in this population.

## Introduction

The treatment of pediatric Inflammatory Bowel Disease (IBD), a chronic, immune-mediated disease, is often complex and can involve various treatment modalities (e.g., medication, surgery, nutrition) (Conrad & Rosh, 2017; Fuller, 2019). Successful treatment of pediatric IBD may involve surgery, including ostomy surgery where a part of the intestine is surgically diverted and stool is collected into a bag worn on the abdomen (Conrad & Rosh, 2017; Fuller, 2019). In pediatric IBD, ostomy surgery may be temporary or permanent depending on the patient’s disease subtype and medical needs (Conrad & Rosh, 2017; Fuller, 2019).

While this remains an emerging area of research across pediatric and adult populations, ostomy surgery is known to be a complex medical decision with potential psychosocial considerations (e.g., body image, anxiety) (David et al., 2016, 2018, 2020, 2023; Fehmel et al., 2018; Nicholas et al., 2008; Savard & Woodgate, 2009). In previous work assessing knowledge of ostomy surgery in pediatric outpatients with IBD, notably few patients (12.5%) endorsed knowledge of this surgery (David et al., 2020); this is concerning as pediatric patients with IBD may undergo ostomy surgery with limited understanding of the surgical intervention until they are facing the decision. Ostomy surgery remains stigmatized across the medical and non-medical communities (Danielsen et al., 2013; Frohlich & Zmyslinski-Seelig, 2016; Rademacher, 2018; Smith et al., 2007), which may also impact healthcare professionals (HCPs) feeling comfortable having conversations about this surgery with pediatric IBD patients and vice versa. Ostomy-related stigma has a complex and intersectional etiology, and research continues to demonstrate this stigma’s negative impact on patient care experiences (Chandhrasekhar & McGee, 2023; Miller & Peck, 2022).

It is hypothesized that patient/family, HCP, and system-level factors shape medical decision-making (MDM) and education of ostomy surgery in pediatric IBD (David et al., 2022), which highlights the multifactorial considerations in improving delivery of developmentally appropriate knowledge to patients/families and how these complex medical decisions are made (David et al., 2023). MDM is defined as the process of integrating education about treatment options and engaging in decision-making about available treatment options to identify a decision (Lipstein et al., 2015; Olszewski & Goldkind, 2018). In this paper, education related to ostomy surgery is defined as education provided to the patient/family that offers information about risks, benefits, outcomes of the procedure, as well as education about logistical and lifestyle considerations (e.g., how to order ostomy supplies, changing the ostomy appliance).

Ostomy-related MDM is complex, and it is critical for HCPs to employ a developmental approach (David et al., 2023; Lipstein et al., 2015; Olszewski & Goldkind, 2018). The medical decision of ostomy surgery also intersects with multiple disciplines and arguably requires nuanced coordination and collaboration between these disciplines for optimal MDM and educational support. Pediatric patients with IBD often interface with multidisciplinary HCPs, including their gastroenterology (GI) medical provider (e.g., physician or advanced practice nurse), surgeon, ostomy nurse, GI social worker, and GI psychologist, across the perioperative timeline (David et al., 2023). There may be notable variation to when, where, and how pediatric patients with IBD interact with HCPs and this can depend on institutional resources, available specialties, and if the surgery is elective or emergent (David et al., 2023). As noted above, ostomy surgery is known to be complex for patients/families with limited current understanding of the HCP and system factors that may impact perioperative MDM and care for these patients. Consequently, exploration of potential HCP and system factors is essential to incorporate into future research and interventions. The current work seeks to better understand the perspectives of multidisciplinary pediatric HCPs on perioperative MDM and education in pediatric patients with IBD considering ostomy surgery in order to identify barriers and facilitating factors to MDM and education for these complex medical decisions.

## Methods

This IRB-approved qualitative study involved cross-sectional, semi-structured, virtual focus groups moderated by psychologists on the research team (JGD, LM). The semi-structured interview script was iteratively developed by members of the research team and based on the current state of ostomy literature (please see references Chandhrasekhar & McGee, 2023 through Miller & Peck, 2022 in the Reference section) and clinical expertise. The semi-structured interview script was designed to elucidate conversation and insights about the perioperative MDM and educational processes in the population of interest. The semi-structured interview script is available upon request to the corresponding author. Multidisciplinary HCPs across five disciplines (GI medical provider, surgeon, GI social worker, ostomy nurse, and GI psychologist) were recruited at a large, urban pediatric medical academic center via email based on established HCP groups (e.g., the GI medical provider email group); these disciplines represent the multidisciplinary specialists who interact with IBD patients across the perioperative spectrum at this institution. Potential participants were informed of the study design, the goal of the study to better understand these concepts in pediatric IBD ostomy care, compensation of a $5 gift card for participation, and the expectation of a one-hour focus group. Inclusion criteria for the study included being a current HCP at the institution, a HCP in one of the five disciplines of interest, having provided care to pediatric IBD patients, and being fluent in English; there were no exclusion criteria.

Sample focus group questions included, “From your perspective and clinical experiences, what do you think are barriers—if any—to deciding about ostomy surgery in pediatric IBD?,” “Please tell me about your perspectives on how medical providers collaborate to make decisions about ostomy surgery in pediatric IBD?,” and “From your perspective and clinical experiences, what recommendations do you have about improving the coordination of multidisciplinary care ostomy education in pediatric IBD?” Focus groups were scheduled to maximize the number of available disciplines present at each focus group. Focus groups were audio-recorded with consent from participants and transcribed via a transcription service (Hoffman Transcription). Transcriptions were analyzed by the moderators who had been present when the focus groups were conducted, using iterative content analysis through a systematic classification process of coding and generating themes with an a priori focus on coordination and collaboration of care until saturation of themes was achieved (Srivastava & Hopwood, 2009).

## Results

Three focus groups of participants recruited via email were conducted and were led by two members of the research team. Focus group 1 included GI medical providers (*n* = 3), GI social worker (*n* = 1), and an ostomy nurse (*n* = 1). Focus group 2 included GI medical providers (*n* = 3) and an ostomy nurse (*n* = 1). Focus group 3 included surgeons (*n* = 2) and a GI medical provider (*n* = 1). No GI psychologists were successfully recruited for the study; of note, 50% (*n* = 2) of the GI psychologists were study authors (JGD, LM) and excluded from recruitment. All participants reported providing care to patients at the institution for an average of 10 years (SD = 9.7), and all worked with pediatric IBD patients to varying degrees. During the course of the study timeline (2020), there were *n* = 9 IBD patients who underwent surgeries for new stomas and *n* = 3 IBD patients who underwent stoma revision surgeries, for a total of *n* = 12 IBD patients who underwent an IBD-related stoma surgery at the authors’ institution.

Two trained members of the research team (JGD, LM) engaged in iterative qualitative analysis, with any code discrepancies resolved through discussion and arriving at a consensus. Through this qualitative analysis, three main qualitative themes emerged from the focus groups. Focus group main themes included (1) HCP perception of ostomies, (2) Patient/family-related factors, and (3) Professional roles and collaboration challenges. Each main qualitative code has associated subcodes, including three subcodes for HCP perception of ostomies, four subcodes for Patient/family-related factors, and four subcodes for Professional roles and collaboration challenges. The qualitative codes, definitions, and participant quotes are captured in Table 1.

**Table 1:** Main qualitative codes and definitions

Main Qualitative Code	Subcode	Definition	Participant quote
Healthcare professional perception of ostomies			
	*Ostomy surgery as a later option for patients*	Healthcare professionals (HCPs) perceiving that ostomy surgery occurred later in care	“It generally is not something that is like a first-line thing….” (GI Physician)
	*“Defeat”/“failure"*	HCPs perceiving that ostomy surgery was a “defeat” or “failure” of other IBD-related treatment from both their own (HCP) perspectives and family’s perspectives	“Generally they get told they failed medical management.” (Ostomy nurse)
	*HCP stigma*	HCPs’ own perceptions of ostomy surgery, including stigma about ostomies, perception that young people will not like/want an ostomy, interest in trying to avoid ostomy surgery as the HCP	“I think there's a pretty big stigma around ostomies, so we talk about it, and so, you know, when we meet with these families and we talk, there's a lot of people out in the world that you don't know have stomas, you know…” (Ostomy nurse)
Patient/family-related factors			
	*Different “categories’” of patients; effort on trying to explain the essential piece of their care (UC versus Crohn’s)*	HCPs’ perspective that the pediatric patient’s IBD subtype and presentation impacts the role of ostomy surgery	“For me it's probably that patients don't like ostomies, so yeah, it's that patients don't like ostomies, so I look at ostomy surgery as, you know, there's one category of patients that I feel that ostomy is sort of mandatory and a vital component of their treatment, and then there's another category of patients…” (Surgeon)
	*Kids feeling unwell medically pre-operatively*	The challenge that young people with IBD are often feeling very unwell before ostomy surgery, which may impact ability to comprehend complex information, engage in medical decision-making, and cope with upcoming surgery	“I think when they're in the hospital, it's obviously a very stressful setting in general, but just like what they said, the patients are really not feeling well at all, and so when you walk in there and you're kind of talking with the families about it, it's just like a different – yeah, like a more acute situation, and it’s hard to have that conversation… or they're just not feeling well, and so I think when I see them in a hospital setting, it can be a little bit stressful, and they're bringing up concerns with school.” (Social Worker)
	*Psychosocial needs/support*	Acknowledgment of the psychosocial needs and support pediatric patients with IBD considering/undergoing ostomy surgery may have, including anxiety/stress in thinking about surgery, worries about peer relationships, body image concerns	“If we had a "Common Ostomy Questions" handout for them or something and to your guys' point about sports, clothes, dance, and activities maybe rather than waiting for them to ask us, we would give it to them proactively…” (GI Physician)
	*Family Considerations/Challenges*	Considerations and challenges with the family system that may impact medical decision-making and education about ostomy surgery from HCPs’ perspectives, including family’s communication style, parent preferences, and patient engagement	“Some of them have like more pointed questions that they don't feel that their child is really going to be even thinking about… You know, ‘Are they still going to be able to have children?.’” (GI Nurse Practitioner)
Professional roles and collaboration challenges			
	*Professional role issues/HCP Role consideration*	The consideration of the roles for multidisciplinary programs and perception around which HCP manages which tasks (e.g., leading surgical decision-making, teaching a family how to change the ostomy)	“Even having like Psychology involved during those conversations or just having someone from like that standpoint could be there to help the medical team even afterwards if you have a family like taking in that information… I think that might be helpful as well when we're talking about language and how to interpret it.” (Social Worker)
	*HCP Concerns/Needs*	The concerns and needs of multidisciplinary HCPs from their perspectives, including concerns for more knowledge, worry about if family will be open to talking about ostomy surgery, and HCP discomfort in talking about ostomy surgery	“You're always balancing that scaring them and saying it's too early and it's not going to happen versus just bringing it up… As docs and practitioners, it would help to become comfortable.” (GI Physician)
	*Timing Considerations/Concerns*	Considerations and concerns related to the timing of medical decision-making about surgery and surgery occurring, including timing of beginning education about ostomy surgery, logistical concerns related to timing of surgery (e.g., surgery on a Friday when a patient is admitted through the weekend), challenges in managing post-operative considerations (e.g., restarting biologic medication)	“Remember a couple times in the past that we did this, consulted, you know, somebody called early and it was still unclear what the plan was, and the family was going back and forth whether they wanted the consult or not, and I think they were offered that, and they either didn't want to talk to us or they took the information, you know, and it was somebody that was doing fairly well on medical management, but the next step would be a stoma, and they kind of smile and nod, take the information, whether they wanted really to see us or not, but, you know, the more times we can reach out to them, I think that's better than preop and maybe two visits postop.” (Ostomy nurse)
	*Coordination/collaboration challenges*	Challenges associated with the coordination and collaboration of the multidisciplinary team, including the importance of the multidisciplinary team, concern for mixed messages across HCPs, variability in coordination/collaboration with psychosocial professionals, logistical coordination concerns across specialties, time, and setting (e.g., inpatient, admitted over the weekend when some services are not on campus)	“I think probably the surgeon probably describes at least what the stoma is, where it's at. We probably do a fair amount in terms of what the stoma should look like. We have the pictures. We actually all have stoma pins on our jackets… you know, to give them an idea of what roughly things will look like. I don't know how much and how thoroughly the GI team. I don't know, you know, not being on that side of it. I think kind of what they will look like on the outside of their belly is probably more the surgeon.” (Ostomy nurse)

*HCP(s)* Healthcare professional(s), *UC* Ulcerative colitis

## Discussion

The decision to consider ostomy surgery in pediatric IBD is often a complex decision that requires nuanced MDM conversations and education for the patient and parent/family (David et al., 2016, 2020). Further, this MDM process often involves coordination and collaboration with multidisciplinary HCPs and can occur across settings (e.g., inpatient, outpatient). This work assessed multidisciplinary HCPs’ perception of MDM and education in pediatric patients with IBD considering ostomy surgery. Qualitative analysis identified three main qualitative themes, including HCP perceptions, patient/family considerations, and professional role and collaboration factors. Notably, the qualitative themes that emerged from this work primarily highlight challenges and barriers; we work to highlight various action steps for HCPs throughout the Discussion to support ameliorating care for our IBD patient populations.

Consistent with previous research in ostomy surgery, the current study noted challenges related to stigma including HCPs identifying their own ostomy-related stigma as well as HCPs perceiving that patients/families carry ostomy-related stigma (David et al., 2023; Frohlich & Zmyslinski-Seelig, 2016; Taft & Keefer, 2016). The intersection between ostomy-related stigma and variable engagement with multidisciplinary team members across the perioperative timeline (e.g., may or may not interact with GI psychology) likely negatively impacts MDM, education, coping, and perception of stigma with ostomy surgery. Further, HCPs’ ostomy-related stigma may impact a patient’s experience of feeling stigmatized, and psychosocial adjustment post-operatively. HCPs’ ostomy-related stigma may impact their language choices in MDM and education (e.g., using stigmatizing terms (“gross”) or assuming that a patient will feel negatively about the ostomy (e.g., “We know patients are scared of ostomies”)) (David et al., 2023). As one participant (surgeon) stated, “For me it's probably that patients don't like ostomies, so yeah, it's that patients don't like ostomies.” Disciplines like GI psychology are likely uniquely suited to support these conversations and reduce stigmatizing language. Participants expressed an interest in support from GI psychology during these conversations with patients and families, though notably this was not described as a currently established practice/standard of care. HCPs are encouraged to reflect on their own ostomy-related stigma and begin to challenge these thoughts and beliefs – where do these messages come from, are there clinical examples that defy these stigmas (e.g., patients with ostomies living full lives), who are these stigmatizing beliefs serving? HCPs can reflect on the language they use in ostomy-related discussions and trial more neutral language, such as avoiding all-or-nothing language. For example, a HCP may say, “Many patients can have questions about swimming with an ostomy…” versus “All patients worry that they cannot swim with an ostomy”). Lastly, HCPs are encouraged to reach out to the psychosocial clinicians through their institutions and/or other resources (e.g., ImproveCareNow’s Social Work and Psychology Group) to ask for interprofessional support in learning more “soft skills” (e.g., active listening, providing empathetic statements) (Lluch et al., 2021) to help reduce stigma in these clinical discussions. In all, there should be a call to action for pediatric psychologists to support IBD surgical medical decision-making and educational processes.

Qualitative analysis identified diverse perspectives on which HCP(s) are perceived by their colleagues to be central to MDM and education processes during the consideration of ostomy surgery in pediatric IBD. Some participants identified the importance of the GI medical provider who likely has the most rapport with the family in managing the young person’s IBD and consequently may be well positioned to engage in ostomy MDM and education. Other participants described their perspectives that the surgeon would be most well positioned to work with families in their MDM and education processes given that the surgeon will ultimately perform the ostomy surgery. In addition, participants also expressed an interest in joint discussions between GI medical providers and surgeons to facilitate conversations about ostomy-related MDM and education. Participants described current and significant logistical barriers to making joint discussions between GI medical providers and surgeons’ the standard of care. In all, challenges related to which HCP(s) begin, lead, and have conversations with patients and families about ostomy surgery exist and may inadvertently contribute to limited optimization of the multidisciplinary team and their collective expertise. For example, a GI medical provider who perceives that ostomy-related MDM and education should be led by the surgeon may defer providing education within their competency to the patient/family (e.g., reassuring families that patients with ostomies can live full and meaningful lives) and also may defer on engaging GI psychology to proactively support ostomy-related coping until the surgeon has initiated the conversation. This may consequently delay an in-depth discussion of surgery and limit opportunities for pre-operative educational interventions and MDM. Participants proposed a multidisciplinary IBD surgery clinic where all disciplines could collaboratively see IBD patients to better facilitate MDM and education along the perioperative spectrum and provide timely and integrated care (e.g., restarting biologic medication after surgery). The clinical coordination for pediatric patients with IBD considering ostomy surgery would strongly benefit from clarification on the roles to ultimately support the patient and family, as well as optimize collaboration across multidisciplinary HCPs. Recent work has demonstrated how multidisciplinary care in pediatric IBD surgical patients reduced emergency room visits and hospital readmissions, which provides encouraging insights into benefits of multidisciplinary care for this population (Short et al., 2022). While the development of multidisciplinary IBD surgery clinics are highly encouraged by the authors, and we also recognize that this recommendation is not accessible to all institutions and this section highlights growth opportunities more equitably available to HCPs. HCPs may take various steps in highlighting the role of multidisciplinary perioperative care, including sharing recent publications with colleagues and department leadership (David et al., 2023), inviting multidisciplinary HCPs to department meetings to speak about how their specialty role can support surgery care (e.g., asking a GI psychologist to present to GI medical providers on psychology’s role in perioperative care), and serving as a physician champion in highlighting and celebrating examples of multidisciplinary care (Dawson et al., 2023).

Participants identified the variability in experiences that pediatric patients with IBD may have depending on factors like the day of the week surgery is done on (e.g., surgery on a Friday where some services like GI psychology and ostomy nurses are not readily available on weekends) and variable coordination and collaboration across multidisciplinary HCPs. As one participant (ostomy nurse) stated, “Depending on when surgery was, that could be, you know, they have surgery on Friday, they're ready to go home Monday, and we’re not here on the weekends at the present time.” This may speak to the patient becoming the constant in their own care and interacting with multidisciplinary HCPs in a non-standardized way at present. In addition, this also likes highlights clinical coordination and collaboration challenges as the pediatric patient with IBD moves between the outpatient and inpatient setting (David et al., 2023). While there is interest around multidisciplinary pediatric IBD ostomy surgery care, challenges related to logistical coordination, communication, and collaboration pose significant barriers and require ongoing research and quality improvement efforts. These coordination challenges in multidisciplinary care have been previously identified (Matlow et al., 2006), including in specialties like palliative care (Mitra & Vadivelu, 2013). HCPs should consider tracking recent IBD ostomy surgery patients through their institution to identify which services these patients interacted with and potential future opportunities for improved communication and collaboration. This is particularly critical in the inpatient setting given the degree of rotating HCPs (e.g., GI medical providers, surgeons) and the need to build reliable systems that are not dependent on specific individual HCPs. For example, this may involve an order set on a surgery service for a pediatric psychology consult to increase integration of psychology support for IBD patients following ostomy surgery. HCPs are also encouraged to facilitate logistical coordination in planning ostomy surgery such as sending a secure handoff to other multidisciplinary services to facilitate collaborative perioperative care (e.g., sharing specific concerns that a family has about the child showering with an ostomy for the ostomy nurse to discuss).

In reviewing study findings, it is important to note limitations to consider, including the institution’s resource/multidisciplinary team and including not having a GI psychologist participate. Participants of the present study work at a singular institution with an established multidisciplinary team and, at the present time, a dedicated GI social worker and two GI IBD psychologists for this IBD subspecialty. It is important to consider that while the participants describe notable challenges in the coordination and collaboration for ostomy surgery in pediatric IBD, other institutions, and HCPs may have different or fewer resources such that this work may not be directly applicable to each institution. While recruitment of a GI psychologist was attempted, ultimately the research team was not successfully in recruiting and representing this perspective in the present work; as there was a participant who is a GI social worker, the research team feels that the present work remains an accurate representation of multidisciplinary work and psychosocial considerations.

The medical decision to undergo ostomy surgery in pediatric IBD is complex and would benefit from multidisciplinary coordination and collaboration for MDM and education. This work sought to understand multidisciplinary HCPs’ perspectives on ostomy-related MDM and education, and identified several qualitative themes across topics like stigma in ostomy surgery, family considerations and preference, logistics of coordinating care, HCP preferences, and diverse perceptions on which HCP leads the discussion. Although all multidisciplinary HCPs have skills and knowledge to uniquely contribute to MDM and education about ostomy surgery in pediatric IBD care, this work suggests notable room to improve and standardize engagement with the multidisciplinary team across the perioperative spectrum. Future clinical and quality improvement work should pursue developing resources that can foster multidisciplinary care (e.g., coping with ostomy pouch changes handout) and interprofessional educational tools to support the ongoing learning of HCPs (e.g., how to reflect, identify, and challenge one’s own stigmatizing beliefs about ostomies). Future research should seek to understand the potential relationship between coordination/collaboration of multidisciplinary care in ostomy surgery in pediatric IBD and medical and psychosocial outcomes (e.g., sustained remission, return to school/activities, body image). This work highlights the developmental and psychosocial considerations in supporting patients and parents through a complex medical decision.

## Data Availability

The data reviewed in this manuscript will be shared after a reasonable request is made to the corresponding author (Dr. Jennie David).
